# Rituximab Induces Complete Remission of Proteinuria in a Patient With Minimal Change Disease and No Detectable B Cells

**DOI:** 10.3389/fimmu.2020.586012

**Published:** 2021-02-08

**Authors:** Maximilian Webendörfer, Linda Reinhard, Rolf A. K. Stahl, Thorsten Wiech, Hans-Willi Mittrücker, Sigrid Harendza, Elion Hoxha

**Affiliations:** ^1^ III. Department of Internal Medicine, University Medical Center Hamburg-Eppendorf, Hamburg, Germany; ^2^ Institute of Immunology, University Medical Center Hamburg-Eppendorf, Hamburg, Germany; ^3^ Institute of Pathology, University Medical Center Hamburg-Eppendorf, Hamburg, Germany

**Keywords:** minimal change disease, rituximab, CD20^+^ T cells, B cell depletion therapy, nephrotic syndrome

## Abstract

Minimal change disease (MCD) is a common cause of nephrotic syndrome. Treatment with steroids is usually effective, but frequent relapses are therapeutic challenges. The anti-CD20 antibody rituximab has shown promising results for treatment of steroid-sensitive nephrotic syndrome. Since predictive biomarkers for treatment efficacy and the accurate rituximab dosage for effective induction of remission are unknown, measurement of CD19^+^ B cells in blood is often used as marker of successful B cell depletion and treatment efficacy. A male patient with relapsing MCD was successfully treated with rituximab, but developed relapse of proteinuria 1 year later, although no B cells were detectable in his blood. B and T cell populations in the patient’s blood were analyzed before and after treatment with rituximab using FACS analysis. Rituximab binding to B and T cells were measured using Alexa Fluor 647 conjugated rituximab. We identified a population of CD20^+^ CD19^−^ cells in the patient’s blood, which consisted mostly of CD20^+^ CD3^+^ T cells. Despite the absence of B cells in the blood, the patient was again treated with rituximab. He developed complete remission of proteinuria and depletion of CD20^+^ T cells. In a control patient with relapsing MCD initial treatment with rituximab led to depletion of both CD20^+^ B and T cells. Rituximab induces remission of proteinuria in patients with MCD even if circulating B cells are absent. CD20^+^ T cells may play a role in the pathogenesis of MCD and might be a promising treatment target in patients with MCD.

## Introduction

Minimal change disease (MCD) is responsible for 10–25% of all cases of a nephrotic syndrome in adults (1). The exact pathomechanisms of MCD remain elusive. However, a circulating factor, most probably secreted by immune cells, is assumed to lead to effacement of podocyte foot processes, leakage of the glomerular filtration barrier, and development of a nephrotic syndrome (2). T cells have been suggested to contribute to the development of MCD (1). The role of the adaptive immune system in MCD is also supported by the presence of functionally impaired regulatory T cells in these patients (3, 4). In addition, the association of reduced regulatory T cells in patients with relapse of the disease (5–7) and the altered transcription regulators reported in B and T cells of MCD patients (8) suggest a role of the adaptive immune system in this disease. This assumed immune-mediated pathogenesis of MCD is the rationale why patients with this disease are treated with immunosuppressive drugs (1, 9).

Steroids lead to remission of proteinuria in 75–80% of adult MCD patients, but relapses occur in up to 56–76% of all cases (1, 10–12). Frequent relapses, steroid-dependence, or steroid-resistance require repeated courses of treatment (9). Thereby, high doses and long-term steroid treatments are needed, frequently leading to adverse effects and toxicity (1, 13). Therefore, alternative immunosuppressive treatments are applied in these patients, including alkylating agents, calcineurin inhibitors, mycophenolate mofetil, and rituximab (1, 10). Rituximab is a mouse-human chimeric anti-CD20 antibody, which induces direct cell death, complement dependent cytotoxicity, and antibody-dependent cell-mediated cytotoxicity in CD20 expressing cells (14, 15). The membrane protein CD20 is a B cell marker and is expressed in human B cells at different stages of their development (16, 17). On the other side, CD20 is not expressed on human podocytes (18). Depletion of CD20 expressing cells using rituximab has shown promising results in the treatment of MCD (10, 13, 19, 20), leading to the hypothesis that B cells have a pathogenetic role in MCD. Nonetheless, the precise mechanisms of action of rituximab in the treatment of MCD are unknown (9, 21). Measurement of CD19^+^ B cells in the blood is used to assess successful B cell depletion, but stable remission has been observed in some patients despite reconstitution of CD19^+^ B cells (22). Recently, the reconstitution of memory B cells but not total CD19^+^ B cells has been shown to correlate with a shorter time to MCD relapse (21). Deciphering the role of both B and T cells in MCD is an ongoing challenge in the understanding of the pathomechanisms of MCD.

Here, we present the case of a patient with MCD, who developed relapses of proteinuria and was successfully treated with rituximab, despite having no detectable CD19^+^ B cells in the peripheral blood. CD20^+^ T cells, which were present in the blood of this patient prior to rituximab treatment and were depleted afterwards, might account for the therapeutic impact of rituximab in this patient.

## Material and Methods

### Diagnostic Laboratory Results

Proteinuria was quantified by photometric measurement of urinary excreted protein in 24 h urine samples and the urinary protein excretion rate (PER; g/24 h) was calculated. Nephrotic-range proteinuria was defined as PER >3.5 g/24 h according to the Kidney Disease: Improving Global Outcomes (KDGIO) nomenclature (23).

### Isolation of Peripheral Blood Mononuclear Cells (PBMC)

EDTA blood was obtained from the index MCD patient and a control MCD patient before and after rituximab treatment. Blood from a healthy donor was used as a control. PBMC were freshly isolated by Ficoll’s protocol and stored in liquid nitrogen until further use. Briefly, EDTA blood was diluted 1:3 in PBS and layered upon Biocoll separating solution 10 mM HEPES (Biochrom, Berlin, Germany). After gradient centrifugation at 1,000 g for 25 min at 20°C, the PBMC layer was carefully removed and washed twice with cold PBS. Lysis of erythrocytes was performed by hypotonic shock. Afterwards PBMC were stored until further use in 10% DMSO (Sigma Aldrich, St. Louis, MO, USA), 30% FCS (Thermo Fisher Scientific, Waltham, MA, USA), and 60% RPMI1640 (Thermo Fisher Scientific) in liquid nitrogen. The study was approved by the local ethics committee of the chamber of physicians in Hamburg (PV4806) and conducted in accordance with the ethical principles stated by the Declaration of Helsinki. An informed consent was obtained from patients and the healthy donor prior to study inclusion.

### Conjugation of Rituximab

Rituximab was conjugated to the fluorophore Alexa Fluor 647 using the Alexa Fluor 647 Protein Labeling Kit (Thermo Fisher Scientific) according to the manufacturer’s instructions. Briefly, 450 µl of rituximab (1.6 mg/ml) were incubated with 50 µl of AF647 reaction dye and 50 µl sodium bicarbonate (1 M) at room temperature for 70 min. Afterwards, matrix column purification was performed to separate excess fluorochrome from labelled rituximab molecules (RTX-AF647). RTX-AF647 was stored at 4°C until use. For all FACS experiments, RTX-AF647 was used at a final dilution of 1:100 in PBS.

### FACS Analysis

All FACS measurements were conducted using a FACS Celesta (BD Biosciences, Franklin Lakes, NJ, USA). PBMC were thawed rapidly at 37°C. After washing with cold PBS, cells were counted using a Neubauer counting chamber and diluted to desired concentrations. For FACS analysis, PBMC were blocked with human serum for 30 min at 4°C and incubated with a pre-titrated antibody cocktail including RTX-AF647 as well as AF750 (Thermo Fisher Scientific), for live-dead staining. For FACS analysis, in each sample at least 200,000 cells were regularly acquired. If not otherwise indicated, all antibodies were obtained from BioLegend (San Diego, CA, USA). The following antibodies and fluorophores were used: V450 anti-CD27 (clone: M-T271), V500 anti-IgD (clone: IA6-2), BV650 anti-CD3 (clone: OKT3), BV785 anti-CD45 (clone: HI30), FITC anti-CD38 (clone: HIT2; BD BioSciences), PE anti-IgM (clone: MHM-88), PerCP anti-CD4 (clone: L200), PE-Cy7 anti-CD19 (clone: HIB19), AF700 anti-CD8a (clone: HIT8a). PBMC were washed twice and measured using a FACS Celesta (BD BioSciences). Graphical analysis was performed using FlowJo version 10.6.1 (FlowJo, Ashland, OR, USA). Of note, in contour plots not all cells are depicted as single dots. For gating strategy see supplemental information (**Figure S1**).

### Histology and Immunohistochemistry

PAS staining and electron microscopy were performed following standard protocols. For immunohistochemical staining for IgG and CD20, 1–2 µm thin slices from formalin-fixed, paraffin-embedded renal biopsies were deparaffinized and pretreated for 15 min at pH6 and 117°C in the autoclave (for CD20) or with proteinase (protease P-8038, Sigma-Aldrich, St. Louis, MO, USA) at 40°C for 15 min (for IgG) followed by incubation with normal serum (Vector S2000, CA, USA) for 10 min. Primary antibodies for IgG (1:7,500) (mouse monoclonal antibody, 209-005-088, Dianova, Hamburg, Germany) and CD20 (1:2,000) (DAKO M0755, CA, USA) were added for 30 min at 40°C. Bound antibodies were then visualized manually using a standard APAAP protocol.

## Results

### Clinical Case

**Figure 1:**
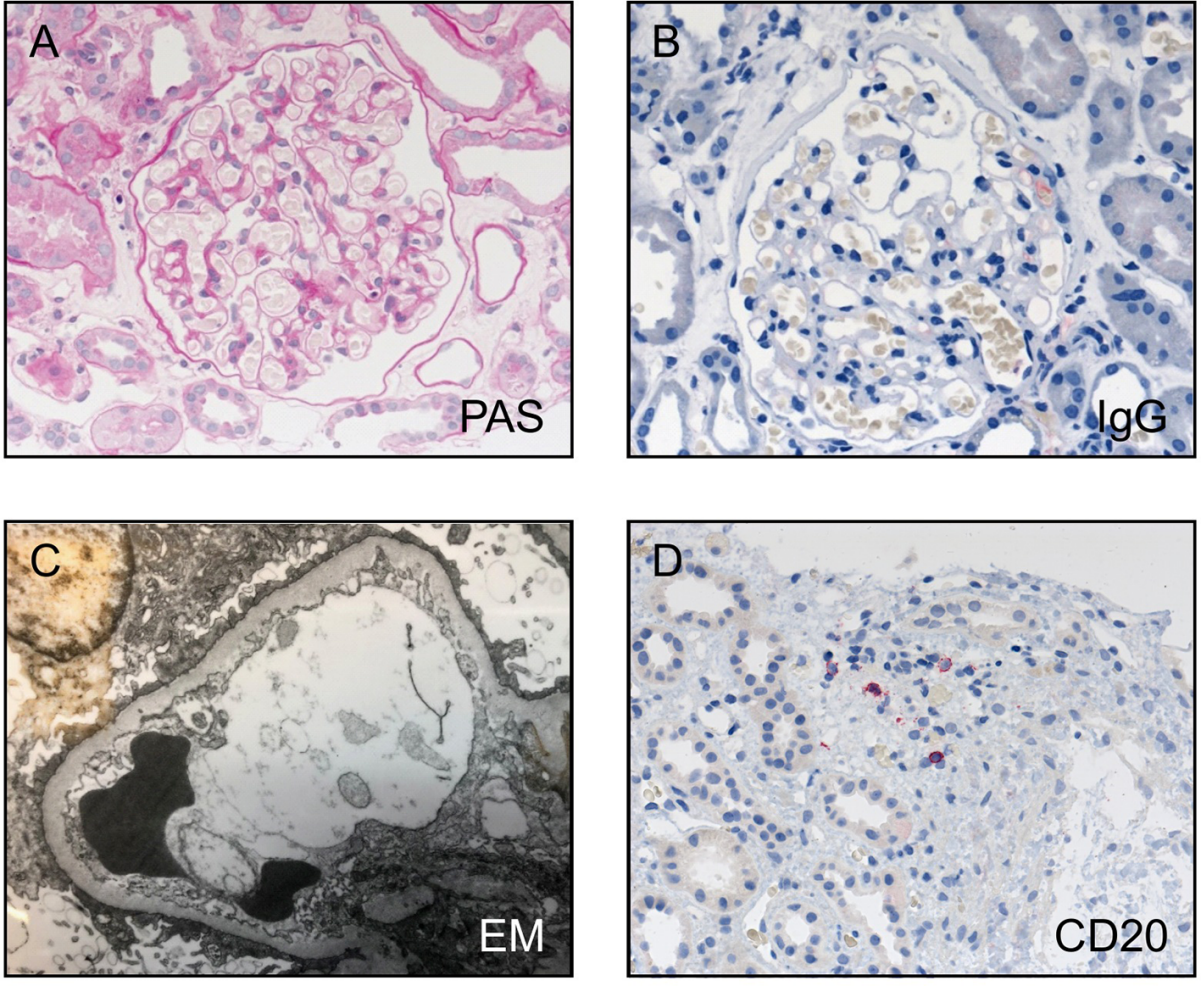
Histological findings in the kidney biopsy of the patient. **(A)** PAS staining, **(B)** IgG staining, and **(C)** electron microscopy confirm the diagnosis of minimal change disease with no IgG positivity, no electron dense deposits, and diffuse loss of podocyte foot processes. **(D)** Only very few CD20 positive cells were detectable, mostly in the area of tubular atrophy and interstitial fibrosis.

A 70-year-old male patient presented at our outpatient clinic with nephrotic syndrome. A kidney biopsy revealed the diagnosis MCD (**Figure 1**). Treatment with steroids resulted in complete remission of proteinuria. However, the patient developed frequent relapses. The first relapse appeared a few months after the initial treatment, while steroids were being withdrawn. Because the patient also had diabetes mellitus type 2 and severe osteoporosis, re-treatment with steroids had to be avoided. He was alternatively treated with rituximab and developed complete remission of proteinuria. In the next 8 years the patient had eight relapses of the nephrotic syndrome, which were all successfully treated with rituximab and the patient went again into complete remission. One year after the last treatment with rituximab, the patient presented again in our outpatient clinic with a relapse of nephrotic syndrome and proteinuria of 8.3 g/24 h. Peripheral blood CD19^+^ B cells were still depleted with a cell count of 1 B cell/µl (normal range 80–500 B cells/µl). Serum creatinine and total leukocyte count in the peripheral blood were within the normal range (1.1 mg/dl and 4,500 cells/µl, respectively). The patient was treated with 1 g rituximab, combined with 60 mg/day prednisolone for 3 days, which was tapered over a time period of 4 weeks, leading to a complete remission of proteinuria within 21 days (proteinuria 0.3 g/24 h).

### Characterization of Peripheral Blood Cells Bound by Rituximab

**Figure 2:**
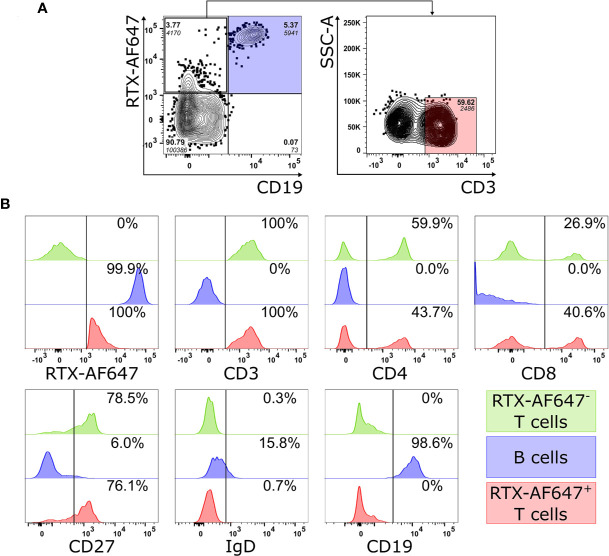
**(A)** FACS analysis of PBMC from a healthy donor gated on CD45^+^ lymphocytes (left panel) shows a population of CD19^−^ RTX-AF647^+^ cells, 59.6% of which expresses the T cell marker CD3 (right panel). **(B)** The expression patterns of RTX-AF647, CD3, CD4, CD8, CD27, CD19, and IgD were assessed in equal counts of CD19^+^ B cells (blue), RTX-AF647^+^ CD3^+^ cells (RTX-AF647^+^ T cells, red), and RTX-AF647^−^ CD3^+^ cells (RTX-AF647^−^ T cells, green). RTX-AF647^+^ T cells (red) express CD3, CD4, CD8, and CD27 at a similar fluorescence intensity as RTX-AF647^−^ T cells (green) and lack the expression of the B cell markers CD19 and surface IgD. For gating strategy see supplemental data (**Figure S1**). Event counts for each gate are indicated in italic below the frequency (bold).

Because of the effect of rituximab treatment on proteinuria in the absence of CD19^+^ B cells in the blood, we aimed to better understand the potential underlying mechanism how rituximab unfolds its effect in this patient. We hypothesized that rituximab targets and depletes CD19^−^ circulating blood cells. Therefore, we first assessed rituximab binding to CD19^−^ blood cells. For this purpose, rituximab was conjugated to the fluorophore AF647 (RTX-AF647) and used to stain circulating CD20^+^ cells. Staining of peripheral CD45^+^ lymphocytes from a 27-year-old male healthy donor with RTX-AF647 led to the identification of CD19^+^ B cells (blue) and a population of CD19^−^ CD20^+^ cells (**Figure 2A**, left panel). A majority (59.6%) of CD19^−^ CD20^+^ cells expressed the T cell receptor associated marker CD3 on the surface. These cells were considered a T cell population (red; **Figure 2A**, right panel) and showed a similar CD3 expression as RTX-AF647^-^ T cells (green), but were enriched for CD8^+^ T cells (**Figure 2B**). RTX-AF647^+^ T cells did not express B cell markers, such as CD19 and surface IgD (**Figure 2B**). CD27 expression was present in 76.1% of RTX-AF647^+^ T cells and 78.5% of RTX-AF647^−^ CD3^+^ T cells, respectively (**Figure 2B**).

### Rituximab Treatment Depletes RTX-AF647^+^ T Cells

**Figure 3:**
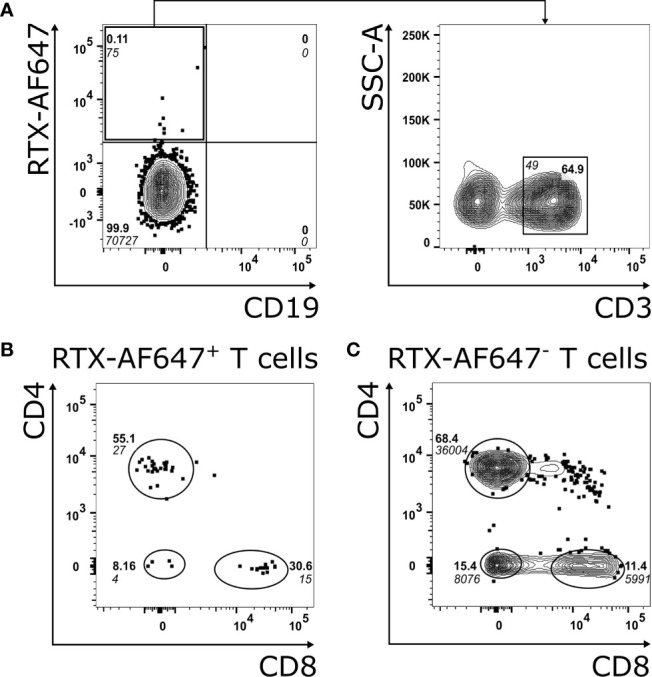
**(A)** FACS analysis of PBMC from the index MCD patient gated on CD45^+^ lymphocytes (left panel) shows a population of RTX-AF647^+^ CD19^−^ cells and no CD19^+^ B cells prior to rituximab treatment. CD3 is expressed on 64.9% of RTX-AF647^+^ CD19^−^ cells (**A**, right panel). **(B)** CD4 and CD8 expression of RTX-AF647^+^ CD3^+^ cells (RTX-AF647^+^ T cells). **(C)** CD4 and CD8 expression of RTX-AF647^−^ CD3^+^ cells (RTX-AF647^−^ T cells). In the blood of the index MCD patient, RTX-AF647^+^ T cells **(B)** are enriched for CD8^+^ T cells and show a reduced frequency of CD4^+^ T cells and double negative T cells in comparison to RTX-AF647^-^ T cells **(C)**.

Next, we analyzed the B and T cell populations in PBMC from the index MCD patient before and after rituximab treatment by flow cytometry. Interestingly, only CD19^−^ RTX-AF647^+^ cells and no CD19^+^ B cells were detected in the blood of the index MCD patient prior to rituximab treatment (**Figure 3A**, left, **Figures S1** and **S2A**). These CD19^−^ RTX-AF647^+^ cells consisted mostly of CD3^+^ T cells (64.9% of RTX-AF647^+^ CD19^−^ cells; **Figure 3A**, right panel). The RTX-AF647^+^ T cell population in the blood of the patient consisted of 55.1% CD4^+^ T cells and 30.6% CD8^+^ T cells (**Figure 3B**), and was therefore enriched for CD8^+^ T cells compared to the total circulating RTX-AF647^−^ T cell population in the same sample, which consisted of 68.4% CD4^+^ T cells and 11.4% CD8^+^ T cells (**Figure 3C**). Noteworthy, in healthy controls, CD4^+^ T cells and CD8^+^ T cells account for 23–52% and 13–40% of the circulating lymphocytes respectively (24). Double negative T cells were found in a lower frequency of 8.16% of RTX-AF647^+^ T cells (**Figure 3B**), while they comprised 15.4% of total RTX-AF647^−^ T cells (**Figure 3C**).

**Figure 4:**
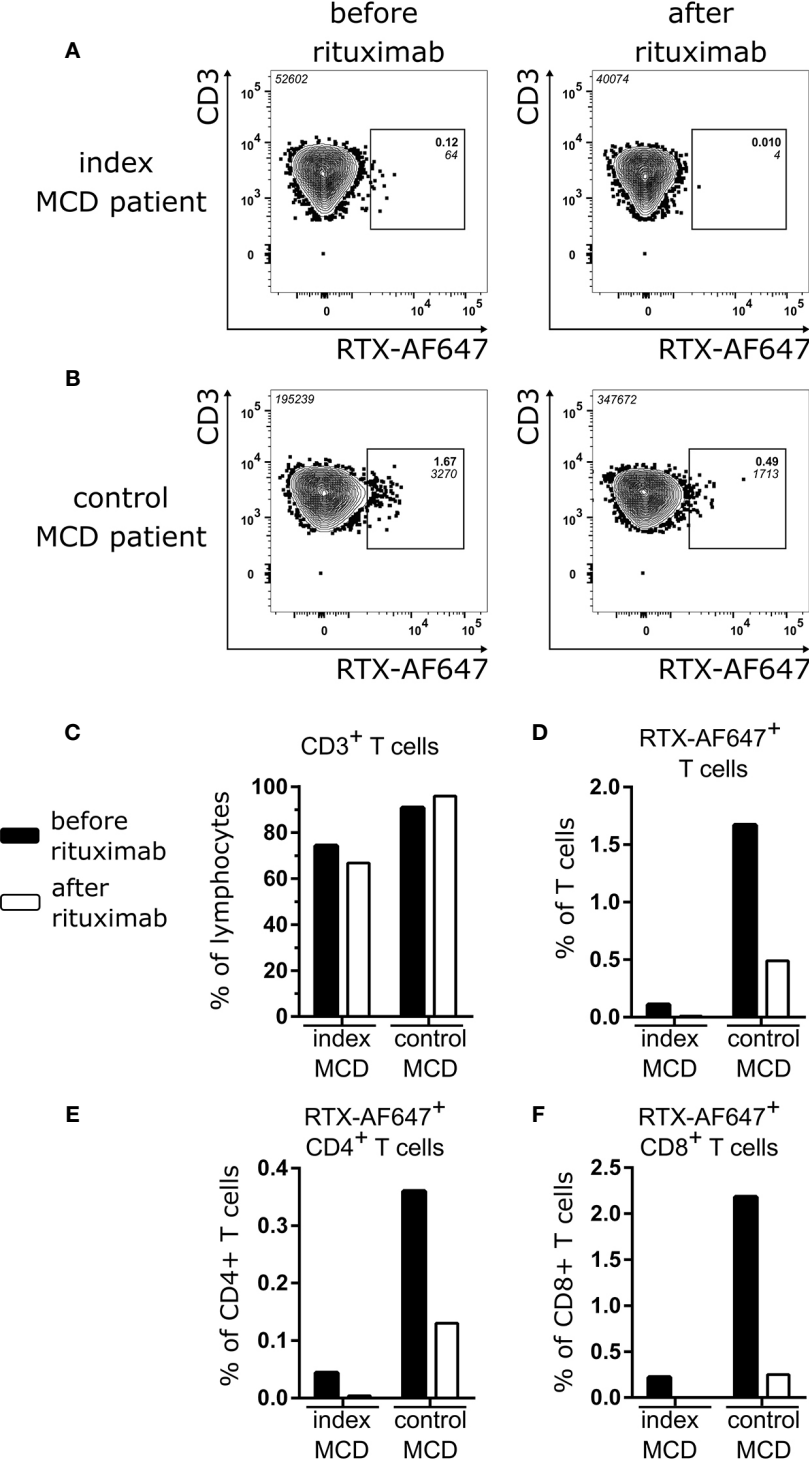
RTX-AF647^+^ CD3^+^ T cells of the index MCD patient **(A)** and the control MCD patient **(B)** before (left panel) and after (right panel) rituximab treatment. **(C)** Frequency of CD3^+^ T cells as percentage of lymphocytes and **(D)** frequency of RTX-AF647^+^ cells among CD3^+^ T cells, **(E)** CD4^+^ T cells, and **(F)** CD8^+^ T cells before (black) and after (white) rituximab treatment in the index MCD patient and the control MCD patient.

Before rituximab treatment, 0.12% of CD3^+^ T cells of the index MCD patient were bound by RTX-AF647 (**Figure 4A**, left panel). After rituximab treatment, the frequency of RTX-AF647^+^ T cells was reduced by 91.7 to 0.01% (**Figure 4A**, right panel).

Next, we aimed to confirm that rituximab treatment leads to depletion of a subpopulation of CD3^+^ T cells in a control patient with relapsing MCD. We performed an identical FACS analysis in a second patient with MCD (control MCD patient). This patient was a 43-year-old female, who was treated for the first time with rituximab. She had both circulating B and T cells prior to rituximab treatment (**Figure S2B**). In the blood of the control MCD patient, we detected RTX-AF647^+^ T cells at a frequency of 1.67% of CD3^+^ T cells prior to rituximab treatment (**Figure 4B**, left panel). After rituximab treatment of the control MCD patient, the frequency of RTX-AF647^+^ T cells was reduced by 70.7 to 0.49% (**Figure 4B**, right panel). Similar findings were made when RTX-AF647^+^ CD19^−^ cells were analyzed. Their frequencies decreased after rituximab from 0.11 to 0.01% in the index MCD patient (**Figure S2A**) and from 1.31 to 0.21% in the control MCD patient (**Figure S2B**), respectively. In the control MCD patient, CD19^+^ B cells were also depleted to a non-detectable level following rituximab treatment, while as shown before, in the index MCD patient CD19^+^ B cells were non-detectable even prior to rituximab treatment (**Figure S2**).

The frequency of CD3^+^ T cells did not significantly change in both the index MCD patient and the control MCD patient after rituximab treatment (**Figure 4C**). The frequency of RTX-AF647^+^ T cells was reduced by 91.7 and 70.7% in the index MCD and control MCD patient, respectively (**Figure 4D**). We further characterized the RTX-AF647^+^ T cell subsets before and after rituximab treatment in both patients. In the index MCD patient CD4^+^ RTX-AF647^+^ T cells were reduced by 91.2% (**Figure 4E**) and CD8^+^ RTX-AF647^+^ T cells were completely depleted (**Figure 4F**). In comparison, in the control MCD patient CD4^+^ RTX-AF647^+^ T cells were reduced by 63.9% and CD8^+^ RTX-AF647^+^ T cells by 88.5% after rituximab treatment.

## Discussion

Frequent relapsing and steroid-dependent MCD patients represent a therapeutic challenge. This is due to the toxicity of long-term steroid treatment. Rituximab is a treatment option for these patients, however, biomarkers to guide therapy are lacking. The B cell marker CD19 is commonly used to predict whether a B cell depleting therapy is a rationale in treating patients with autoimmune kidney diseases. Despite showing no CD19^+^ B cells circulating in the blood, the presented index MCD patient developed a relapse of nephrotic syndrome. Moreover, he developed a complete remission of proteinuria within 3 weeks of treatment with rituximab and steroids. Remission of proteinuria lasted almost 1 year, similar to the remission phases during the last 8 years of treatment with rituximab. As CD19 and not CD20 is commonly used as a B cell marker to follow the depletion of B cells after rituximab, we investigated whether CD19^−^ CD20^+^ cells were present in the patient’s blood, which could be targeted by rituximab and might play a role for disease induction. We were able to characterize a population of CD19^-−^ RTX-AF647^+^ cells in the blood of the patient, which consisted mostly of CD3^+^ T cells.

After rituximab treatment, proteinuria resolved and CD20^+^ T cells were depleted. There are several potential explanations for the relapse of the nephrotic syndrome in the absence of B cells and the successful induction of remission following rituximab treatment. Firstly, pathogenic B cells could have reconstituted without being detectable in the patient’s circulation. Such CD20 positive cells may be resident in the tissue and thus are not detected in the blood. The depletion of these potentially pathogenic B cells may have contributed to the remission of proteinuria. Relapses of nephrotic syndrome have been shown to be associated with reconstitution of memory B cells, which reappear only after naïve B cells are detectable in the circulation (21). In our patient, however, neither naïve nor memory B cells were detected in the blood at the time of relapse. Therefore, cryptic reconstitution of memory B cells seems unlikely. Secondly, B cells could have reconstituted but escaped detection due to technical limitations. However, CD19^+^ B cells were detected successfully in the blood of the control MCD patient and a healthy control using our method of detection. Thirdly, RTX-AF647^+^ T cells could play a role in the relapse of a nephrotic syndrome and the depletion of these CD20^+^ T cells following rituximab treatment may be a relevant therapeutic mechanism.

Contrary to B cells, circulating CD20^+^ T cells were detected at the time of relapse in a low frequency (0.12% of T cells) in the blood of the index MCD patient. In the blood of healthy controls, the mean frequency of CD20^+^ T cells usually varies between 1.6 and 3.8% of CD3^+^ T cells (25, 26). This shows that in our index MCD patient CD20^+^ T cells had already reconstituted after the previous rituximab treatment, in contrast to B cells. As RTX-AF647^+^ T cells were depleted after rituximab treatment in both the control MCD patient as well as the index MCD patient, we conclude that rituximab binds specifically to CD20^+^ T cells and depletes them *in vivo*.

MCD has been considered a T cell mediated disease (27, 28), while the role of B cells remains unclear (21). Nonetheless, CD20 targeted antibody therapies have shown promising results in the treatment of relapsing MCD (7, 10, 19, 29, 30). The removal of autoreactive T cells has been proposed as a therapeutic mechanism of rituximab (31) and treatment of MCD patients with rituximab leads to the depletion of CD4^+^ CD45RO^+^ CXCR5^+^ T cells, invariant natural killer T cells and CD4^−^ CD8^−^ T cells (7, 32). The depletion of CD20^+^ T cells may represent a link connecting the successful application of anti-CD20 antibody treatment and the role of T cells in the pathogenesis of MCD.

Whether T cells are capable of expressing CD20 has been controversial since the first description of CD3^+^ T cells co-expressing CD20 (33). Several studies were able to show the expression of CD20 in T cells on a single cell level by imaging flow cytometry (34, 35), confocal microscopy (36), and immunohistochemistry (37). Together with the endogenous transcription of CD20 mRNA in CD20^+^ T cells (25, 26, 34, 35), this supports the view of endogenous synthesis of CD20 in a subset of T cells. Accordingly, CD20 antibody treatment depletes CD20^+^ T cells in the blood along with B cells (25, 26, 34–36, 38–42). CD20 expressing T cells have been described in several autoimmune diseases, e.g. multiple sclerosis (25, 35, 39, 41, 42), rheumatoid arthritis (26, 36, 43), primary Sjögren’s syndrome (38), and psoriasis (40), as well as HIV infection (34). Our patient is the first case where these cells are reported in an immune-mediated nephrotic glomerular disease.

Clinical data describing the role of CD20^+^ T cells in autoimmune disease are scarce. In patients with psoriasis, frequency of circulating CD20^+^ T cells producing IL-17, IL-21, and TNFα correlates with disease activity (40). Recently, it has been suggested, that CD20^+^ T cells contribute to the pathogenesis of multiple sclerosis and depletion of CD20^+^ T cells may play a role in the therapeutic mechanism of anti-CD20 antibody treatment (42, 44, 45). In patients with relapse of multiple sclerosis, it was shown that after anti-CD20 antibody treatment B cells were hardly detectable in the blood at a time when CD20^+^ T cells were already replenished (25). This finding has similarities to our index MCD patient, who showed reconstitution of CD20^+^ T cells while B cells were still depleted at the time when proteinuria relapsed. Other studies have also shown that CD20^+^ T cells reconstitute months before B cells reappear in the circulation after rituximab treatment (25, 35).

This is the first report describing CD20^+^ T cells in the context of a frequent relapsing nephrotic syndrome in a patient with MCD. Rituximab combined with a short steroid treatment induced remission of proteinuria despite undetectable B cells. We found CD20^+^ T cells in the blood of the patient at the time of relapse, which were depleted after re-treatment with rituximab. Rituximab successfully induced complete remission of proteinuria despite of the absence of CD19^+^ B cells. Patients with relapse of nephrotic syndrome may benefit from rituximab treatment irrespective of the frequency of CD19^+^ B cells. Therefore, low or absent CD19^+^ B cells in the blood might not be used as disconfirming evidence for the effectivity of rituximab for relapse of nephrotic syndrome. More studies are needed to decipher the role of CD20^+^ T cells in autoimmune kidney diseases. This may help to guide clinical decisions for the use of CD20 antibody treatment in kidney autoimmune diseases and may lead to a better understanding of the pathogenesis of MCD.

## Data Availability Statement

The original contributions presented in the study are included in the article and **Supplementary Material**. Further inquiries can be directed to the corresponding author.

## Ethics Statement

Written informed consent was obtained from the individuals for the publication of images and data included in this article.

## Author Contributions

EH, SH, and RS were responsible for the conception and design of the study. MW and LR performed the experiments. H-WM and MW developed the FACS strategy. EH, H-WM, and MW interpreted the FACS data. EH and SH analyzed and interpreted the clinical data. TW analyzed and interpreted the morphological and immunohistochemical findings. Drafting and revising of the article were performed by MW, EH, and SH. All authors contributed to the article and approved the submitted version.

## Funding

This study was supported by a grant from the Deutsche Forschungsgemeinschaft as part of the Sonderforschungsbereich 1192 (project B1 to EH and RS, project A4 to H-WM, project B6 to TW, project C1 to EH and RS) and the Heisenberg Programme to EH.

## Conflict of Interest

The authors declare that the research was conducted in the absence of any commercial or financial relationships that could be construed as a potential conflict of interest.
