# Supplementary material for: Rituximab Induces Complete Remission of Proteinuria in a Patient With Minimal Change Disease and No Detectable B Cells
Source: Front Immunol. 2021 Feb 8;11:586012. doi: 10.3389/fimmu.2020.586012 (PMC7897659; doi:10.3389/fimmu.2020.586012)
Supplement: Supplementary file 1 [file DataSheet_1.docx]

Supplementary Material

# Supplementary Figures and Tables

**
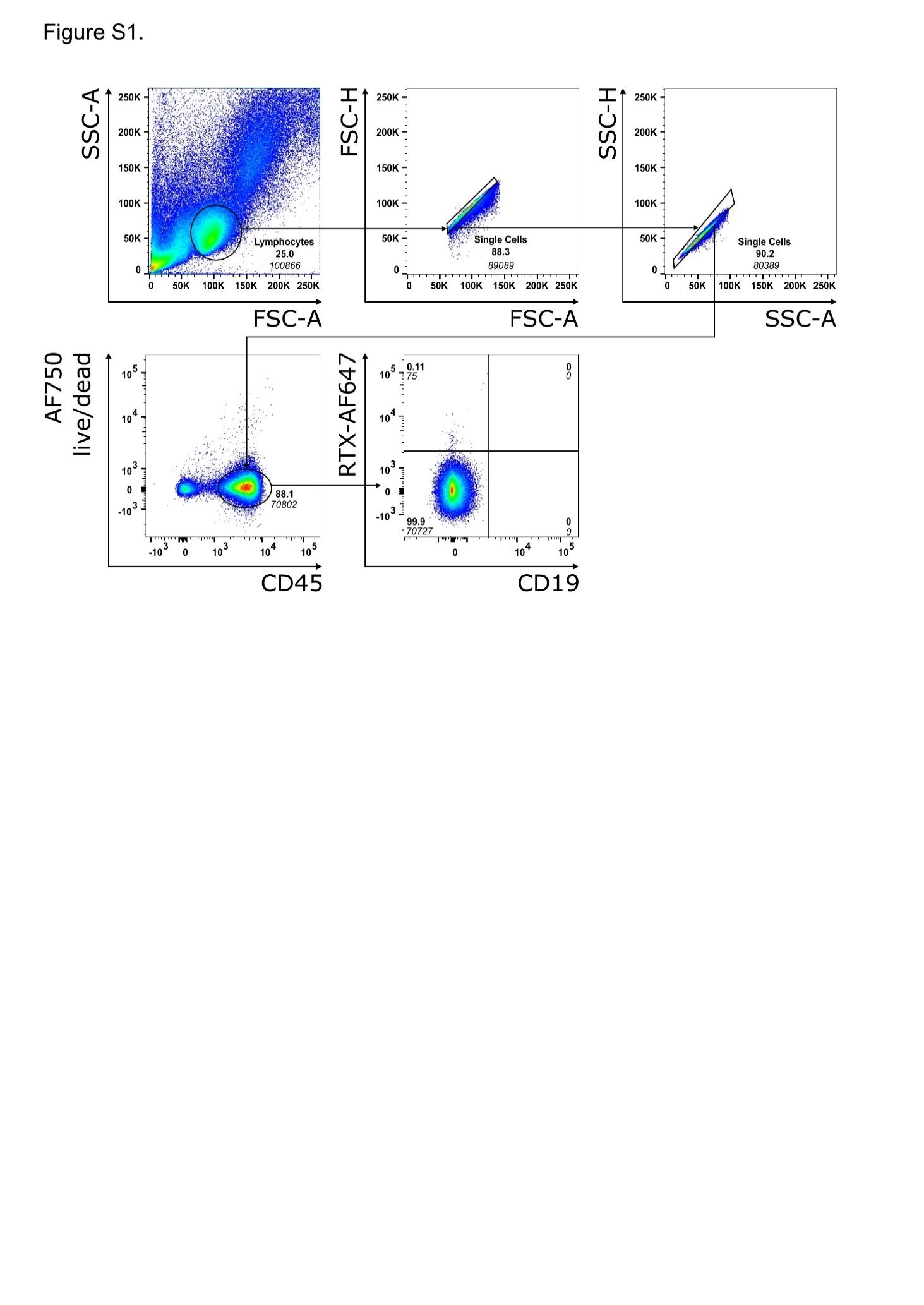
**

**Figure S1.** Representative FACS gating strategy of PBMC from the index MCD patient. After gating on lymphocytes, doublets were excluded using the forward and sideward scatter. Dead cells were excluded using AF750 as a live-dead staining and CD45^+^ lymphocytes were identified. All further gating steps originate from AF750^-^ CD45^+^ lymphocytes, if not otherwise indicated. Event counts for each gate are indicated in italic below the frequency (bold).


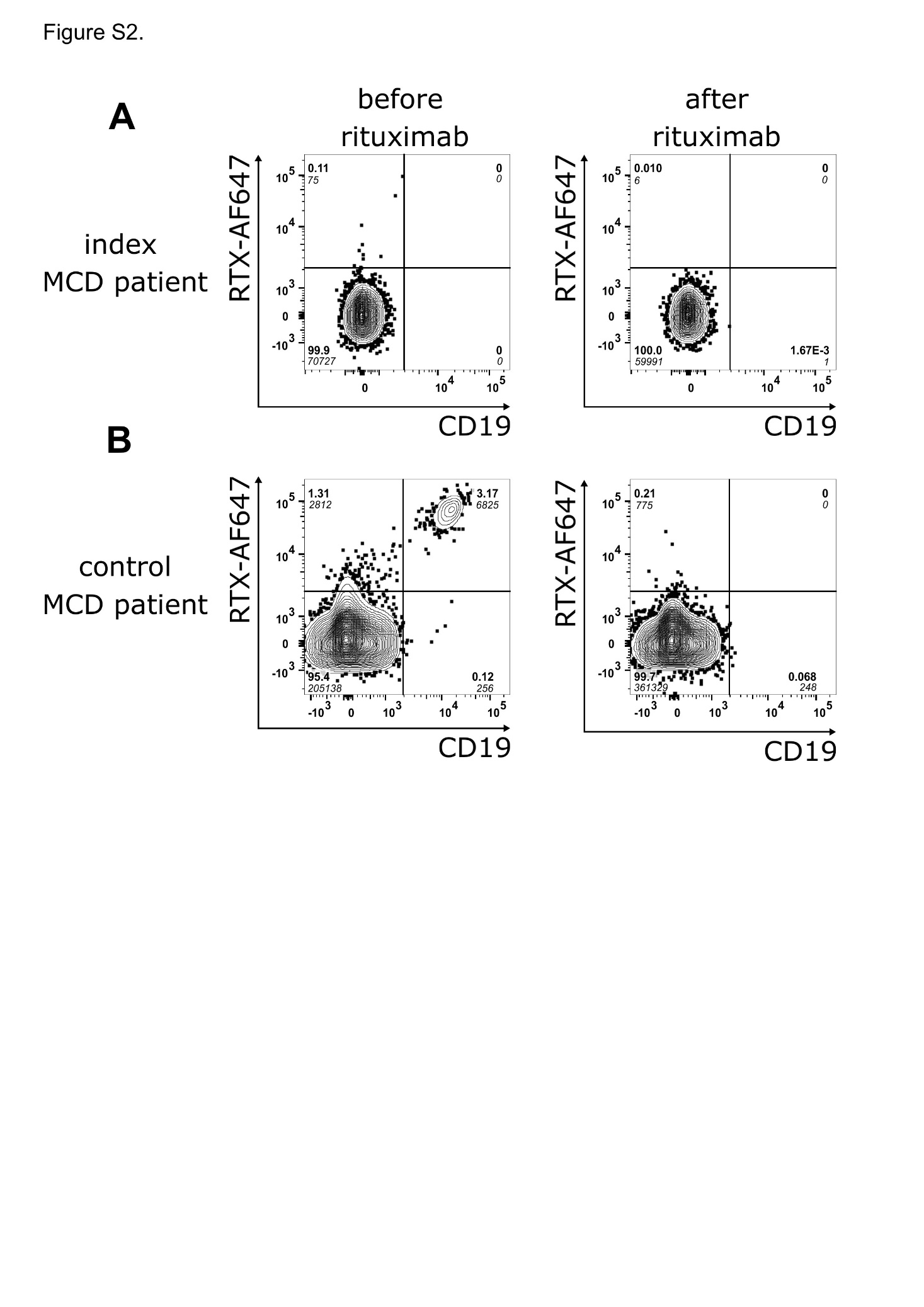


**Figure S2.** FACS analysis of PBMC gated on live CD45^+^ lymphocytes from (A) the index MCD patient and (B) the control MCD patient before (left panels) and after (right panels) rituximab treatment. In the index MCD patient, no CD19^+^ B cells were detected prior or after treatment with rituximab. CD19^-^ RTX-AF647^+^ cells were also depleted after rituximab treatment. In the control MCD patient, both CD19^+^ B cells and CD19^-^ RTX-AF647^+^ cells were depleted after rituximab treatment. Event counts for each gate are indicated in italic below the frequency (bold).
